# Complement Factor C5a Inhibits Apoptosis of Neutrophils—A Mechanism in Polytrauma?

**DOI:** 10.3390/jcm10143157

**Published:** 2021-07-17

**Authors:** Christian Ehrnthaller, Sonja Braumüller, Stephanie Kellermann, Florian Gebhard, Mario Perl, Markus Huber-Lang

**Affiliations:** 1 Institute of Clinical and Experimental Trauma-Immunology (ITI), University of Ulm, 89081 Ulm, Germany; sonja.braumueller@uniklinik-ulm.de (S.B.); steffi-denk@web.de (S.K.); 2 Department of Orthopedics and Trauma Surgery, Musculoskeletal University Center Munich (MUM), University Hospital, LMU Munich, 81377 Munich, Germany; 3 Department of Traumatology, Hand-, Plastic-, and Reconstructive Surgery, Center of Surgery, University of Ulm, 89081 Ulm, Germany; florian.gebhard@uniklinik-ulm.de (F.G.); mario.perl@web.de (M.P.); 4 Department of Traumatology and Orthopaedic Surgery, University Hospital Erlangen, 91054 Erlangen, Germany

**Keywords:** C5a, neutrophils, apoptosis, polytrauma

## Abstract

Life-threatening polytrauma results in early activation of the complement and apoptotic system, as well as leukocytes, ultimately leading to the clearance of damaged cells. However, little is known about interactions between the complement and apoptotic systems in PMN (polymorphonuclear neutrophils) after multiple injuries. PMN from polytrauma patients and healthy volunteers were obtained and assessed for apoptotic events along the post-traumatic time course. In vitro studies simulated complement activation by the exposure of PMN to C3a or C5a and addressed both the intrinsic and extrinsic apoptotic pathway. Specific blockade of the C5a-receptor 1 (C5aR1) on PMN was evaluated for efficacy to reverse complement-driven alterations. PMN from polytrauma patients exhibited significantly reduced apoptotic rates up to 10 days post trauma compared to healthy controls. Polytrauma-induced resistance was associated with significantly reduced Fas-ligand (FasL) and Fas-receptor (FasR) on PMN and in contrast, significantly enhanced FasL and FasR in serum. Simulation of systemic complement activation revealed for C5a, but not for C3a, a dose-dependent abrogation of PMN apoptosis in both intrinsic and extrinsic pathways. Furthermore, specific blockade of the C5aR1 reversed C5a-induced PMN resistance to apoptosis. The data suggest an important regulatory and putative mechanistic and therapeutic role of the C5a/C5aR1 interaction on PMN apoptosis after polytrauma.

## 1. Introduction

Immediately after a traumatic impact, the resulting tissue injury generates numerous danger molecules, so-called damage-associated molecular patterns (DAMPs) [1]. DAMPs in concert with oxygen radicals, neuro-mediators, and cytokines can lead to an early systemic inflammatory response [2]. Depending on the severity of the injury, the injured organism must cope synchronically with additional exogenous danger signals, so-called pathogen-associated molecular patterns (PAMPS) from exogenous and endogenous microorganisms [3]. Innate immunity and in particular neutrophil granulocytes and the complement system can recognize this molecular danger early after injury.

The complement system represents a serine protease cascade that is activated in a step-by-step proteolysis [4]. This process can be initiated by several pathways, namely the classical, alternative, and lectin pathways. In addition, there is also the possibility of complement activation by activated coagulation factors (thrombin pathway) [5], which are excessively produced in the context of severe traumatic injuries. In the converging end, the terminal complement complex (TCC) is formed by C5b-9 complement factors and can act as a cellular signal and ion pore in cell membranes (then defined as membrane attack complex, MAC), ultimately leading to the lysis of the target damaged cell or microorganism [4]. Injured and/or hypoxic tissue as well as endotoxins lead to activation of the complement system with generation of the activation products C3a, C5a, and sC5b-9 as well as the opsonin C3b [6]. The anaphylatoxins C3a and C5a can induce all classical signs of inflammation, including enhanced vascular permeability [7]. In particular, C5a is a very potent chemotactic factor for neutrophil granulocytes and upregulates the expression of key adhesion molecules at the vascular endothelium [8]. In the clinical setting, elevated plasma concentrations of C3a and C5a were found after severe trauma or during sepsis overall correlating with poor outcome [6,9].

Another endogenous system for innate defense against danger represents apoptosis. Programmed cell death can help the clearance of damaged cells but also worsen overall tissue loss. In principle, apoptosis can be initiated by two main activation pathways: The extrinsic pathway is triggered by the death receptor family (e.g., Fas (CD95) or TNF). This leads to formation of the death-inducing signaling complex (DISC) and activation of caspase 8, which in turn activates caspase 3 and initiates apoptosis [10,11]. The intrinsic mitochondrial pathway can be triggered by oxidative stress and various cytokines/chemokines. Through these stimuli, anti-apoptotic factors (such as Bcl-2, Bcl-xL, and McL1) and pro-apoptotic factors (such as Bax, Bad, Bim, Bid, and Bak) are imbalanced, resulting in the formation of the apoptosome and ultimately in programmed cell death [12]. A third activation pathway, the endoplasmic reticulum (ER) pathway, is the least understood and is activated by oxidants and calcium dyshomeostasis [13]. In severely traumatized patients, of note, a delayed apoptosis rate of circulating PMNs was found despite enhanced circulating pro-apoptotic factors [14,15,16]. However, a decreased expression of anti-apoptotic factors within the PMNs and disturbances in the extrinsic pathway were also detected and associated with septic complications [16]. 

The complement and apoptosis system exhibit similarities in their tasks of recognition and final elimination of damaged cells. Although increasing evidence for interactions of the two exist, a deeper understanding is missing. In neutrophils from polytraumatized patients, enhanced concentrations of the pro-apoptotic factor granzyme B have been found, which in vitro could cleave C3 and C5 into C3a and C5a, respectively, independently of the established complement proteases [17]. Experimental studies in rodents with sepsis revealed that excessive C5a plays a decisive role in conferring PMN resistance to apoptosis via the PI-3K/AKT pathway [18]. Here, the blockade of C5a even abrogated the decreased apoptosis [18], indicating a potential crossroad of C5a and resistance to apoptosis. 

In the context of polytrauma, both enhanced C5a production [19] and decreased neutrophil apoptosis [14,16] have been separately demonstrated. However, potential underlying linking mechanisms remained in the dark, which are addressed in the present study.

## 2. Materials and Methods

### 2.1. Patients

Blood samples from healthy volunteers and from polytraumatized patients at different time points after injury were used in this prospective, monocentered, descriptive observational study. Study approval was obtained by the Independent Local Ethics Committee of the University of Ulm (ethic votum No. 69/08), and written informed consent was obtained from the patients and healthy volunteers. Informed consent was obtained from patient’s legal representatives when the patient was unconscious. Ten multiply injured patients admitted to the trauma-I-level Department of Trauma with an ISS ≥ 18 were included in the study, and blood samples (Serum, EDTA, Citrat Monovettes, Sarstedt, Germany) were obtained immediately after admission (0 h) and 4, 12, 24, 48, 120, and 240 h after trauma. Exclusion criteria were age < 18 years, gravidity, cardiopulmonary resuscitation, HIV or TBC infection, recent radiation or chemotherapy, and withdrawal of consent. For some demographic details, see the Supplementary Table S1. Furthermore, blood samples from twelve healthy male donors were drawn. Whole blood was collected using two syringes containing EDTA or the anticoagulant citrate dextrose (1:10; Fresenius Kabi, Germany).

### 2.2. Neutrophil Isolation for In Vitro Characterization

Citrated blood from healthy volunteers was used and PMN was isolated as described elsewhere [20]. In brief, cells were separated by Ficoll–Paque density gradient centrifugation (Pharmacia Biotech, Stockholm, Sweden) followed by a dextran sedimentation step. After hypotonic lysis of residual RBCs, PMN were resuspended in HBSS++. 

### 2.3. Neutrophil Cultivation and Induction of Apoptosis

PMN isolated by Ficoll–Paque centrifugation/dextran sedimentation [20] from healthy male volunteers were adjusted to 2 × 10^6^ cells/mL in HBSS++ (Gibco, Thermo Fisher Inc., Waltham, MA, USA) + 5% FCS (Biochrom, Berlin, Germany), seeded on 24 well-plates, and incubated for 2 h at 37 °C (5% CO_2_) with different concentrations (1–1000 ng/mL) of either human recombinant C3a (Calbiochem, Merck Millipore, Darmstadt, Germany) or human recombinant C5a (Sigma-Aldrich, St. Louis, MO, USA). Apoptosis was induced by stimulation of the extrinsic pathway with an agonistic anti-Fas antibody (Fas-agonistic antibody, CH11, 500 ng/mL, Merck Millipore, Darmstadt, Germany) or the intrinsic pathway with H_2_O_2_ (hydrogen peroxide H_2_O_2_ 0.025% Fischar, Saarbrücken, Germany) in the presence of the low molecular C5aR1 antagonist (10 µg/mL, kindly provided by Prof. John D. Lambris, University of Pennsylvania). For inhibition of the PI3K, the PMNs were preincubated with Wortmannin for 15 min (250 nM, Sigma-Aldrich, St. Louis, MO, USA). 

### 2.4. Quantification of Apoptosis by Flow Cytometry

PMN apoptosis was assessed: (1) in EDTA whole blood directly after being drawn at the defined time points post polytrauma or directly after being drawn from male healthy volunteers, which then was stained with FITC-labeled Annexin V and 7-AAD, lysed by FACS lysing solution (BD Pharmingen, San Diego, CA), washed twice, and fixed (1% paraformaldehyde plus 0.1% sodium azide) (as previously described [21]) followed by flow cytometric analyses; and (2) in isolated PMN (from citrated blood, see above) for the indicated in vitro studies. PMNs were stained for Annexin V/7-amino-actinomycin D (7-AAD) following the manufacturer’s instructions. Briefly, cells were adjusted to 2 × 10^6^ cells/mL in binding buffer and stained with FITC-labeled Annexin V and 7-AAD on ice for 20 min. Cell gating included separation of the granulocytes and monocytes by the typical forward and side light scatter profiles and additional staining with anti-CD45/anti-CD14 (Leukogate; Beckman Coulter, Kreefeld, Germany). Fluorescence measurement was performed in both cases on a FACSCanto™ II flow cytometer using FACSDiva software v9.0 (BD Biosciences, Franklin Lakes, NJ, USA). Cells were classified as viable (Annexin Vlow/7-AADlow), early apoptotic (Annexin Vhigh/7-AADlow), and late apoptotic or necrotic (Annexin Vhigh/7-AADhigh). 

### 2.5. PMN Surface FasR and FasL Expression

EDTA blood from healthy volunteers and polytraumatized patients drawn at the indicated timepoints after trauma was used. Cells in EDTA whole blood were stained with a PE-labeled anti-FasL and PE-labeled anti-Fas antibody (Bio-Rad, Hercules, CA, USA) for 20 min. After lysis of red blood cells with FACS Lysing Solution (Cat. Nr. 349292, BD Biosciences, Franklin Lakes, NJ, USA), cells were resuspended in DPBS, and fluorescence measurement was performed on a FACSCantoTM II flow cytometer using FACSDiva software v9.0 (BD Biosciences, Franklin Lakes, NJ, USA).

### 2.6. PMN Surface C3aR and C5aR1 Expression

To determine C3aR and C5aR1 expression on neutrophils, EDTA whole blood from heathy volunteers and polytraumatized patients in the dynamic course was obtained and processed as previously described [21]. In brief, blood was incubated with FITC-labeled anti-C3aR (Bio-Rad, Hercules, CA, USA) or anti-C5aR1 (CD88, Bio-Rad, Hercules, CA, USA) or corresponding FITC-labeled isotype-matched IgG for 20 min. After lysing of the erythrocytes by FACS lysing solution for 10 min and two washing steps, the leukocytes were fixed and analyzed by flow cytometry (FACSCantoTM II).

### 2.7. Serum FasL and sFasR ELISA

EDTA anti-coagulated blood collected from healthy volunteers and polytraumatized patients was centrifuged at 800× *g* for 5 min; the supernatant was centrifuged at 13,000× *g* for another 2 min to pellet cellular debris, and supernatants were stored at −80 °C until further analysis. FasL and sFasR concentrations in samples were analyzed using DuoSet ELISAs (R&D Systems Inc., Minneapolis, MA, USA) following the manufacturer’s instructions.

### 2.8. Caspase 3/7 Activity Quantification

Apoptosis was induced in PMN isolated from healthy male volunteers as described above. After an incubation time of 2 h at 37 °C (5% CO_2_), caspase 3/7 activity was assessed by the Caspase-Glo^®^ 3/7 Assay (Promega, Madison, WI, USA) following the manufacturer’s instructions. Chemiluminescence was measured using a Luminometer (Luminoskan Ascent; Thermo Scientific; Life Technologies Holding Pte. Ltd., Singapore).

### 2.9. Apoptosis Array

After the induction of intrinsic and extrinsic apoptosis at 37 °C (5% CO_2_) for 2 h with and without the additional administration of C5a (100 ng/mL), the subsequent lysis of PMN was performed, and the cell lysate was used for proteome analysis. For the detection of apoptosis-related proteins, a commercially available membrane-based antibody array was used strictly in accordance with the manufacturer’s protocol (ARY009 Proteome Profiler Assay, R&D Systems Inc., Minneapolis, MN, USA).

### 2.10. Statistics

All values are expressed as mean ± standard error of the mean (SEM). After testing for normal distribution by the Kolmogorov–Smirnov test, datasets were analyzed using one-way analysis of variance (ANOVA) followed by the Dunnett method as a post hoc test. In case of nonparametric distribution, Kruskal–Wallis one-way analysis of variance on ranks was performed followed by Dunn’s method. The statistical software was Sigma Plot 14.0 (Systat Software Inc., San Jose, CA, USA). Results were considered statistically significant when *p* < 0.05.

## 3. Results

### 3.1. Decreased Apoptosis of PMN and Alterations of the FasL–FasR–Axis after Polytrauma

**Figure 1:**
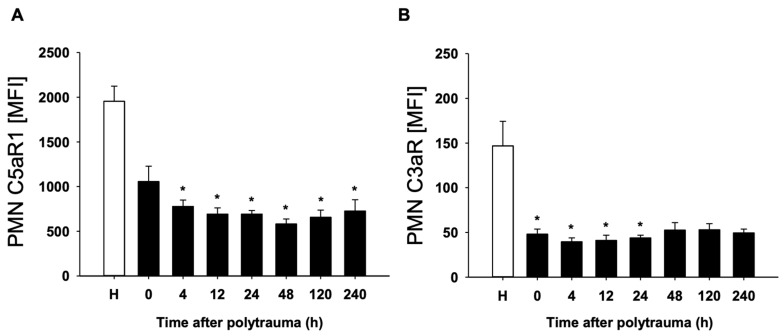
Mean fluorescence intensity (MFI) of C5aR (**A**) and C3aR (**B**) on PMN of polytraumatized patients (*n* = 10) in hours versus healthy controls (H). (*n* = 12). Results are shown as mean ± SEM. * = *p* < 0.05 vs. healthy volunteers.

In a first step, complement activation was assessed by systemic appearance of the complement activation products C3a and C5a in the blood and by reduction of the corresponding PMN expression of the C3aR as well as C5aR1. As expected, enhanced levels of C3a and C5a were measured (as early as at admission: C3a: 143 ± 17 ng/mL after polytrauma versus 71 ± 23 ng/mL in healthy probands and C5a: 1.7 ± 0.2 ng/mL after polytrauma versus 1.2 ± 0.2 ng/mL, respectively). Furthermore, a significant reduction of both anaphylatoxin receptors, C3aR and C5aR1, on PMN surfaces was found by flow cytometry (Figure 1A,B), indicating systemic complement activation post injury.

**Figure 2:**
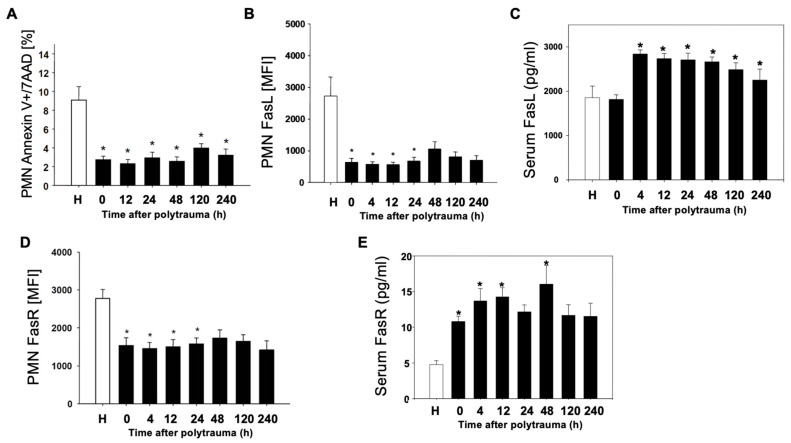
Reduced PMN apoptosis after polytrauma. Time-course of apoptotic events in PMN (*n* = 10) post trauma vs. healthy donors (*n* = 12). (**A**): Annexin positive, 7AAD negative PMN of polytraumatized patients in percentage in the post-traumatic time course; (**B**,**D**): Mean fluorescence intensity (MFI) of FasL and FasR on PMN of polytraumatized patients in hours versus healthy controls (H). (**C**,**E**): Serum FasL/FasR in pg/mL in serum of polytraumatized patients. Results are shown as mean ± SEM. * = *p* < 0.05 vs. healthy volunteers.

In order to find out whether cell-specific regulation of the apoptosis rate after polytrauma takes place in parallel to complement activation, the PMN apoptosis rate as well as Fas-ligand and Fas-receptor on the surface of leukocyte populations after polytrauma were determined. Already at the time of admission to the emergency room (0 h), a significant reduction of the apoptosis rate was evident in PMN (Figure 2A). Furthermore, there was an early and sustained decrease in FasL on PMN (Figure 2B). In contrast, a significant increase in serum FasL was detected (Figure 2C). Analysis of FasR also showed a significant and sustained decrease on the surface of PMN (Figure 2D) and a significant increase in serum FasR (Figure 2E).

### 3.2. Anaphylatoxin C5a Inhibits PMN Apoptosis

**Figure 3:**
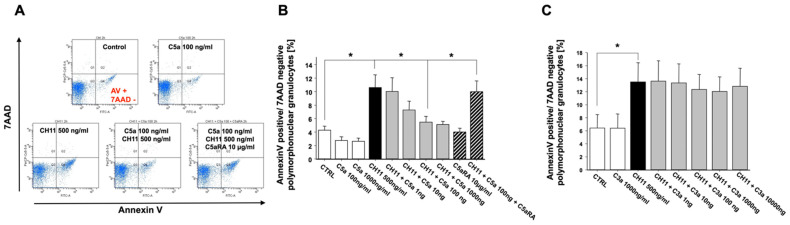
Inhibition of the extrinsic, CH11 (CD95)-induced apoptosis rate of freshly isolated PMN from healthy volunteers by exposure to C5a (concentration–response curve) in the absence or presence C5aR1 antagonist (C5aRA) (*n* = 4) (**A**: original flowcytometric recording; **B**: summary). (**C**) Similar conditions under C3a exposure (*n* = 3). The percentage of apoptotic PMN was determined by Annexin-V/7-AAD staining and flow cytometry. Results are shown as mean ± SEM. * = *p* < 0.05.

In further studies, the influence of the central complement activation products C3a and C5a on alteration of the apoptosis rate of PMN from healthy donors and its regulation was investigated. The presence of C5a could inhibit the FasR-mediated extrinsic apoptotic pathway (by the activating antibody CH11) in PMN in a dose-dependent manner (Figure 3A,B). Remarkably, these inhibitory effects were completely reversible upon cellular exposure to a specific small-peptide inhibitor of the C5aR1 (PMX53). However, the addition of C3a did not alter the apoptotic rate, even not at higher concentrations (Figure 3C).

**Figure 4:**
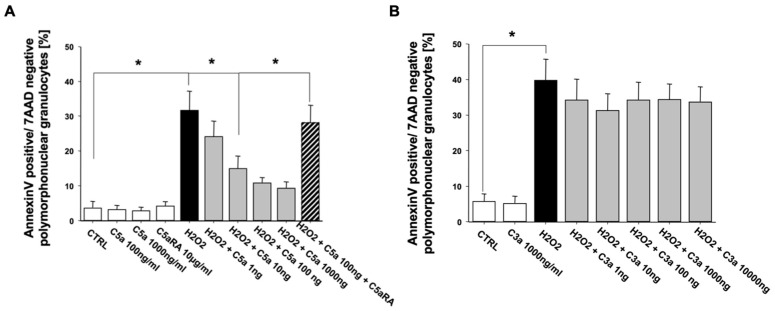
C5a (*n* = 4) (**A**) but not C3a (*n* = 3) (**B**) inhibits the intrinsic, H_2_O_2_-induced apoptosis rate of freshly isolated PMN from healthy male volunteers in a concentration-dependent manner. The percentage of apoptotic PMN was determined by Annexin-V/7-AAD staining and flow cytometry. C5aRA = C5aR1 specific small peptide inhibitor PMX53. Results are shown as mean ± SEM. * = *p* < 0.05.

Similar to the results of extrinsic apoptosis in PMN, C5a also exerted a concentration-dependent inhibition of the intrinsic PMN apoptosis pathway (H_2_O_2_-induced). Again, this protection against intrinsic apoptosis was completely abrogated under C5aRA treatment (Figure 4A). In contrast, C3a was not able to exert such effects addressing the intrinsic pathway (Figure 4B).

**Figure 5:**
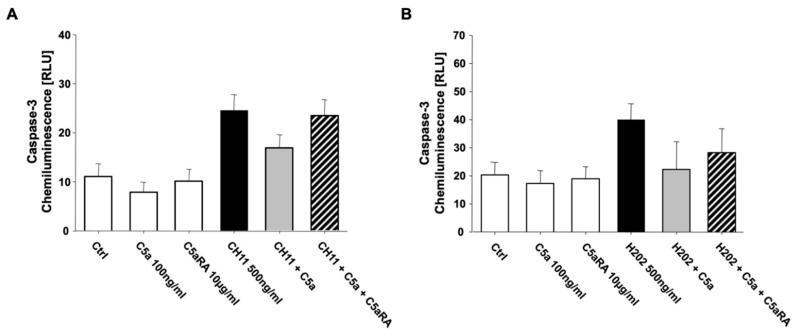
Caspase-3 activity measured by a chemiluminescence assay of (**A**) extrinsic CH11- (*n* = 3) as well as (**B**) intrinsic H_2_O_2_-induced apoptosis in freshly isolated PMN from healthy male volunteers (*n* = 3). C5aRA = C5aR1 specific small peptide inhibitor PMX53. Results are shown as mean ± SEM.

Analysis of the key caspase-3 activity in extrinsic as well as intrinsic apoptosis under C5a exposure showed matching tendencies with the Annexin V/7-AAD measurement, although these tendencies were not significant (Figure 5A,B). Both extrinsic as well as intrinsic apoptosis was abolished after administration of the pan caspase inhibitor Z-VAD (data not shown).

**Figure 6:**
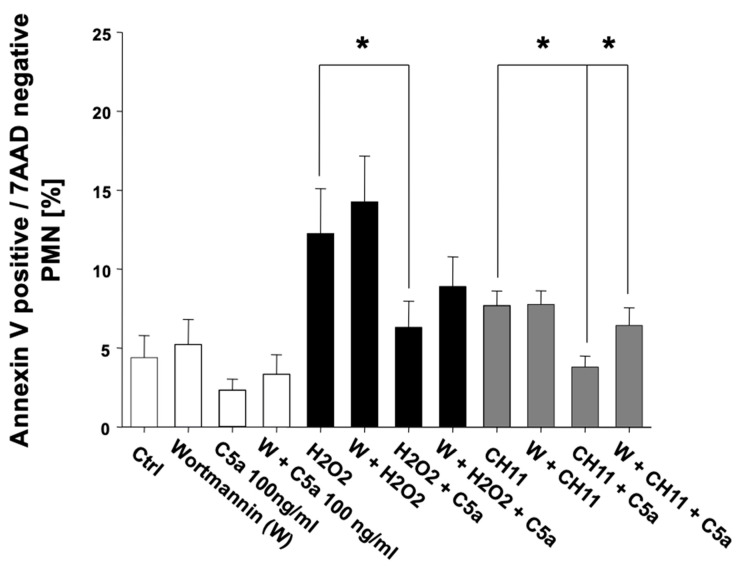
Inhibition of PI3-kinase by wortmannin (W) results in a partial reversal of the C5a-caused reduction of PMN apoptosis. Comparison of the apoptosis rate of freshly isolated PMN from healthy male volunteers (*n* = 5) in percent under influence of C5a and W before (transparent) as well as after induction of intrinsic, H_2_O_2_-induced (black) as well as extrinsic, CH11-induced apoptosis (gray). The percentage of apoptotic PMN was determined by Annexin-V/7-AAD staining and flow cytometry. Results are shown as mean ± SEM. * = *p* < 0.05.

Thus, C5a inhibited both intrinsic- and extrinsic-induced apoptosis of PMNs. These effects were apparently C5a-specific, as C3a intervention did not alter the apoptosis rate of PMN. Further complementary studies focused on the possible signal transduction pathways involved. A proteome profiling of PMN lysates after C5a exposure addressing several key apoptotic factors and regulators failed to reveal significant changes in the protein concentrations (Figure S1). Additional Western blot analyses of lysates from PMN after incubation with or without C5a and apoptosis induction (with H_2_O_2_ or CH-11) revealed no significant alterations of MCL-1 Bax, Bim, PUMA, Noxa, and BCL-2 (data not shown). However, addition of the PI3K inhibitor wortmannin resulted in partial inhibition of the C5a-protective effect with respect to PMN apoptosis (Figure 6).

## 4. Discussion

Several studies have demonstrated reduced or delayed apoptosis in PMNs after both experimental and clinical polytrauma, even in the presence of high serum levels of pro-apoptotic factors [14,15,22,23]). Furthermore, the degree of apoptosis resistance after traumatic injury also correlated with clinical scores for the development of organ failure [14].

Mechanistically, the increased life-span of PMN and activated complement during the systemic post-traumatic response [19] may both help clear debris and microbes—but it can also attack host cells and drive barrier dysfunction with subsequent (multiple) organ failure [2]. In the present study, we confirmed a significant decrease in the apoptotic rate of PMN over a time course of up to 10 days after polytrauma. Several factors have been proposed to cause enhanced apoptotic resistance of neutrophils during inflammatory conditions including acute phase protein α-1 antitrypsin [24], the anti-apoptotic Bcl-2 [16], and lipopolysaccharids [25]. It has also been proposed that the complement activation product C5a can confer PMN resistance to apoptosis. Using much higher, unphysiological concentrations (ug range) of C5a and much longer time intervals (up to 24 h) in comparison to the more physiological conditions used in the present study, a reduced apoptotic rate was demonstrated involving to some extent the phosphoinositide 3-kinase (PI3K) [26] and extracellular-signal regulated kinases (ERK) [27] pathway. Previous in vitro analysis with unphysiological C5a concentrations (1 ug/mL) revealed CREB (cAMP response element-binding protein)-mediated Bcl2 transcription as the underlying pathway for C5a-mediated apoptosis resistance [28]. In the present study, already early inhibitory effects of PMN apoptosis (within 2 h) upon stimulation of the C5a–C5aR1 axis (in more physiological concentrations: 10–100 ng/mL) could be detected. In the context of polytrauma, an early generation of C5a has been reported by several groups [29] and could easily provide the anaphylatoxic microenvironment and C5a concentrations in a 10–100 ng/mL range for the PMN to become more resistant against apoptotic processes. Moreover, our group previously showed first evidence of cross-talking complement-apoptosis systems after trauma: the pro-apoptotic granzyme B within the leukocytes was enhanced after polytrauma and capable of cleaving C5 to C5a, which then can obviously exhibit anti-apoptotic features [17]. Further evidence for an apoptosis–complement interplay is evidenced by the fact that apoptotic cells display a decreased expression of endogenous complement inhibitors and thus can increase complement-mediated elimination [30].

It is noteworthy that the role of the complement in general differs depending on the targeted cell and underlying disease. For example, two decades ago, the blockade of C5a was shown to reduce apoptosis of thymocytes in sepsis [31]. The same group demonstrated that PMN in sepsis increased again by treatment with a C5a antagonist, thus leading to normal apoptosis rates [18]. Therefore, delayed apoptosis of PMN in the context of polytrauma does not necessarily translate to reduced apoptotic events in other circulating or organ cells.

In the present study, the significant decrease in the PMN apoptosis rate was associated with a sustained decrease in FasL and FasR on PMN and a corresponding increase in FasL and FasR in serum up to 10 d after injury, which was possibly due to early, trauma-associated release of FasL and also of FasR from cell surfaces into blood. These findings are coherent with previous studies highlighting a major role in PMN apoptosis for FasR-signaling [11,32,33]. In accordance, another study showed a positive correlation of FasR serum levels with organ dysfunction during sepsis and suggested FasR as a possible target for the prevention of hyperinflammation in sepsis [34]. The latter researchers also evaluated PMN apoptosis after major trauma and sepsis development in more detail and found the activation of both intrinsic and extrinsic apoptosis pathways to be inversely correlated with disease outcome [16]. Therefore, in the present study, the intrinsic and extrinsic paths were examined in more detail in regard to complement activation. Both intrinsic and extrinsic apoptosis showed a significant concentration-dependent inhibition under the influence of C5a as early as 2 h after exposure. This effect was completely reversible with the addition of a specific C5aR1 antagonist. Remarkably, although related in molecular structure, no similar effects could be shown under the influence of the central anaphylatoxin C3a. Therefore, a specific role for the C5a–C5aR1 axis regarding apoptosis impairment in PMN could be one possible reason. Other studies demonstrated the differential effects of complement activation on apoptosis. In a PMN-like cell line, a dual function of the C5aR, anti- and pro-apoptotic, was proposed: the ribosomal protein S19 dimer (RP S19, generated by apoptotic cells) interacts with the C5aR, leading to an accumulation of regulators of G protein signaling 3 (RGS3) and downregulation of ERK, finally resulting in pro-apoptotic effects [35]. In contrast, the C5a molecule competitively inhibited the RP S19–C5aR interaction and thereby upregulated ERK signaling, resulting in anti-apoptotic effects [35].

As previous studies demonstrated that the BCL-2 family member MCL-1 serves as an upstream regulator of PMN apoptosis [36] and MCL-1 upregulation could be diminished after treatment with FasL [16], further characterization was undertaken. However, a proteome array approach and Western blot analyses failed to indicate a significant role for anti-apoptotic MCL-1 and BCL-2 or pro-apoptotic Bax, Bim, PUMA, and Noxa after exposure of PMN with C5a. In the further search of underlying pathomechanisms, previous cell studies of untreated PMN suggested that the PI3K pathway, among others, appears to be especially important for signal transduction in regard to apoptosis and C5a [26]. In respect to the aforementioned study, the present work demonstrates conclusive data showing PI3K-dependent apoptosis regulation of C5a. It is tempting to speculate that protective effects of C5a could at least be partially abrogated in both intrinsic and extrinsic apoptosis by addition of the PI3K inhibitor wortmannin. Since C5a can induce PI3K-dependent phosphorylation of Akt [37], this downstream signaling could theoretically also be involved. However, not only the PI3K pathway seems to have effects on complement-induced survival of PMN. Further studies by Perianayagam showed that the ERK pathway also leads to the phosphorylation of Bad and thus to the inhibition of apoptosis [27], and that C5a furthermore increases transcription of the anti-apoptotic BCL-2, which was modulated by CREB [28].

Our results indicate that the anaphylatoxin C5a, shown by several groups to be systemically generated early after polytrauma [29], seems to prolong PMN life-span both via the intrinsic and extrinsic pathway. 

As a limitation of the study, a pharmacological inhibition of C5a was—although effectively—only performed in vitro but not in vivo after (poly)trauma. However, the latter only would be capable of directly linking altered C5a concentrations after trauma to altered apoptosis resistance of the PMNs. It also remains speculative whether C5a-caused prolonged PMN life-span in the context of polytrauma will prove beneficial or detrimental for the clinical course and final outcome. Furthermore, it remains to be clarified in a larger trauma patient cohort whether and to which extent slightly enhanced systemic C5a or reduction in the corresponding anaphylatoxin receptors directly correlates with decreased apoptosis rates of PMNs.

Putting this study in a larger perspective, enhanced generation of the anaphylatoxin C5a may not only moderate apoptotic events in the in vitro setting or extreme setting of polytrauma but also in other diseases such as infections, ischemia (e.g., myocardial infarction or stroke), neurodegenerative diseases, and even in cancer, because in all these conditions, a local or systemic complement activation has been described [38,39]. Therefore, it will be worthwhile to decipher the exact mechanistic cross-link of the anaphylatoxin C5a and the molecular switch for delayed apoptosis, and in this regard, ohmic approaches will likely be helpful. Concerning the detected post-traumatic loss of the C3aR along with the C5aR1 on PMN, this could be further developed as a monitoring tool, as for the C5aR1 in critically ill patients, the loss of C5aR on PMN correlated with an enhanced risk for nosocomial infections [40] and development of multiple-organ failure [41]. To what extent the C3aR on PMN can be utilized as a monitoring tool in other easy-to-access cells (e.g., monocytes or lymphocytes) remains to be elucidated. Although the present study did not show an in vitro modulation of apoptosis of PMN by C3a, C3aR seems crucially involved in the neuro-inflammatory response [42] and thus should also be tested in other tissues, organs, and diseases for apoptosis-modulating features.

## Data Availability

The data presented in this study are available on request from the corresponding author. The data are not publicly available due to ethical and privacy policy reasons.

## Supplementary Materials

The following are available online at https://www.mdpi.com/article/10.3390/jcm10143157/s1, Figure S1: Apoptosis Proteome Profiler Assay of PMN lysates 2 h after exposure to C5a or control conditions. N = 3; results are shown as mean ± SEM. * = *p* < 0.05, Table S1: Demographic data of the enrolled polytrauma patients.
