# Supplementary material for: Complement Factor C5a Inhibits Apoptosis of Neutrophils—A Mechanism in Polytrauma?
Source: J Clin Med. 2021 Jul 17;10(14):3157. doi: 10.3390/jcm10143157 (PMC8303460; doi:10.3390/jcm10143157)
Supplement: Supplementary file 1 [file jcm-10-03157-s001.zip › jcm-1247486-supplementary.pdf]

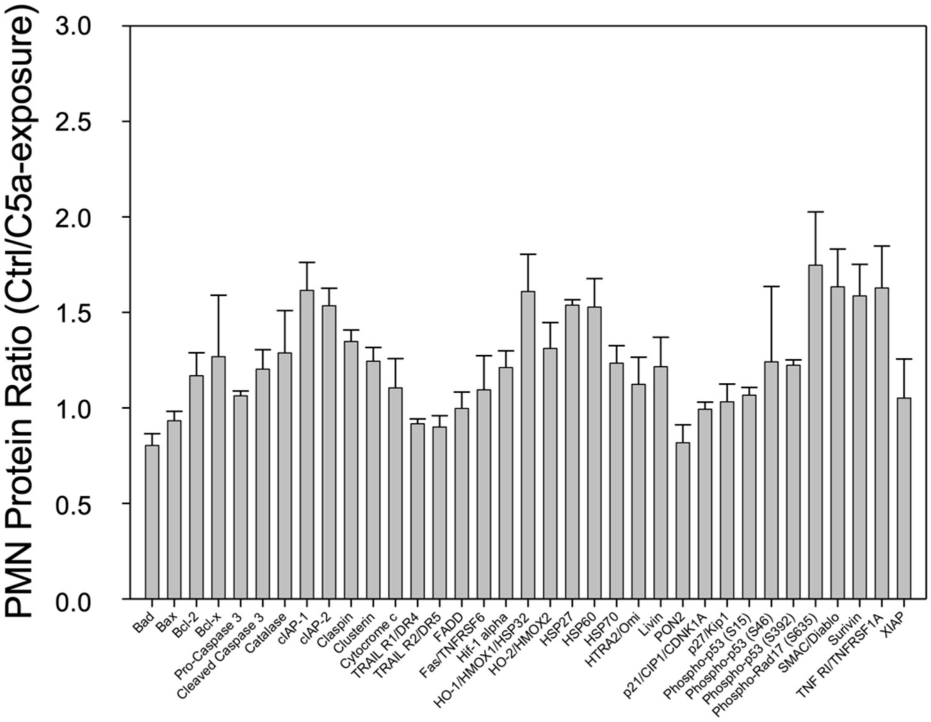

Figure S1. Apoptosis Proteome Profiler Assay of PMN lysates 2 h after exposure to C5a or control conditions. N = 3; results are shown as mean ± SEM. * = $p < 0.05$.

Table S1. Demographic data of the enrolled polytrauma patients.

Parameter	Mean	+/− SEM
Age (y)	51.0	6.9
Male: Female (n: n)	8:2	n/a
Trauma Etiology (vehicle accident: work-related: home)	5:3:2	n/a
Injury Severity Score (ISS)	28.5	2.8
Glasgow Coma Scale (GCS)	9.8	1.4
Traumatic Brain Injury (n/10)	7/10	n/a
Thorax Trauma (n/10)	8/10	n/a
Abdominal Injury (n/10)	2/10	n/a
Pelvic Fracture (n/10)	4/10	n/a
Fractures of the Extremities (n/10)	6/10	n/a
Base Excess (Admission) (mmol/L)	−0.79	1.07
Transfusion (packed red blood cells) within 12 h post trauma	8.5	2.2
Interleukin-6 at Admission (pg/mL)	133	40
Survival Rate (30 d Observation Period) (n/10)	9/10	n/a
